# Causal ACTH-Depot Therapy during Pregnancies following Infertility Treatment

**DOI:** 10.1155/2012/248926

**Published:** 2012-05-15

**Authors:** Rudolf Klimek, Marek Klimek, Peter Gralek, Dariusz Jasiczek

**Affiliations:** Fertility Centre Cracow, Plac Szczepański 3, 31-011 Cracow, Poland

## Abstract

The aim of this paper was to confirm the efficacy of adrenocorticotropin depot (ACTH-depot) therapy in pregnancies with threatened miscarriage and preterm delivery through the desired stimulation of the adrenal glands controlled by the rest of organism. The activity of hypothalamic-pituitary-adrenal axis plays a key role in pregnancy. Such naturally stimulated endogenous corticosteroid hormones are free from unwanted side effects of their synthetics analogs. Low level of maternal blood ACTH and insufficient increase of induced by hypothalamic hormones oxytocinases (cystine-**β**-aminopeptidases) were indication to ACTH-depot therapy (0.5 mg/week) in our consecutive prospective studies. Contrary to antenatal use of synthetic corticosteroids, there are no temporal limits of this therapy, which has to be more often recommended into clinical prevention of fetal morbidity, treatment of premature delivery, and finally elimination of the newborn's mortality caused by the neuroendocrinological gestoses.

## 1. Introduction

Hormonal therapy during pregnancy must take into consideration the state of both the mother and the fetus. This applies to the indications on the mother's side (e.g., diabetes, thyroid, and adrenal gland diseases) and the child's side (intrauterine development disorder) and to the dynamically changing relationship between these two organisms. During pregnancy, there is also an increase in the production of hormones and enzymes of the placenta, the function that has an essential meaning in the mutual mother-fetus neuro-immunoendocrine relationship [1–4]. This applies especially to the synthesis and concentration adjustment of isooxytocinases (cystine-beta-aminopeptidase (CAP_1_) and isocystine-beta-aminopeptidase (CAP_2_)), which decompose hypothalamic hormones [5–7]. Any damage to the placenta (partial separation, calcification and vascular clots), or only hypoxia, leads to a decrease of the concentration of these enzymes in the mother's blood, which automatically results in the increase of not only oxytocin and vasopressin but also corticotropin-realising hormone (CRH) and gonadotropin-realising hormone (GnRH), which, in consequence, provokes a change in the production of steroid hormones, essential for the pregnancy. In 1957, Tuppy and Nesvadba described the principles of chemical assay of the enzyme oxytocinase using as substrate L-cystine-di-*β*-naphthylamide [8]. The following years brought an increasing interest in assessment of aminopeptidase activity in obstetrics, but in 1966 Babuna and Yenen published a study on the modification of the original chemical assessment of oxytocinase that in fact hindered investigations of enzyme monitoring in pregnancy [9]. Three years later, these laboratory methods were corrected, but they were published in a chemical journal, which was readily available for clinicians [10]. However, the introductions of new methods of pregnancy monitoring as well as divergence of opinion on the application of oxytocinase, resulted in overshadowing of the study on cystine-aminopeptidase activity in the pregnant women. The oxytocinases reflect the state of the mother, the fetus, and the placenta. Maternal blood levels of CAP_1_ and CAP_2_ show high correlation with the fetal and placental mass as well as with the fetal maturity state [11]. The constant increase of oxytocinasemia up to the time of delivery in 81% of pregnancies and potential stabilization of its level during the last weeks of pregnancy in a further 12% of cases are the most sensitive indicators of the proper development of fetuses. In the case of the hormone deficient neurosecretion in hypothalamic nuclei and/or insufficiency of the placenta, the blood level of the enzyme is low, its physiological increase is stopped, or the concentration is even lowered. In women with hypothalamic insufficiency syndromes that have not been diagnosed in time, intrauterine death of the fetus occurs in at least 50% of cases, for which replacement hormonal therapy with adrenocorticotropin of prolonged activity (ACTH-depot) is successful [3, 12].

 Modern techniques of monitoring and treatment of pregnancy as a coexistence of mother and child gained a new dimension through the individual technical quantization of the fetal maturity process and in it the discovery of a decisive role of two axes: hypothalamic-pituitary-adrenal axis and hypothalamic-pituitary-gonadal axis. These axes are under the influence of pregnancy stimuli, from the change in the size of the uterus to the synthesis of CRH in the mother, fetus, and the placenta, since the mechanical stimulation of the mammary glands or the pressure of the speculum on the vagina stimulates the production of hormones in the hypothalamus [11, 13–16].

 Medical intervention must firstly take into consideration the natural process of gestation. When using synthetic hormones, one must remember that such medications contain an additional mixture of residual side products of their industrial manufacture. For example, the omission of one atom of nitrogen and hydrogen in an oxytocin molecule doubles the increase in hormonal activity but at the same time blocks the possibility of effective action of the next dose of thus-created desaminooxytocin. It is the exercise of the possible action upon the receptors of dexamethason or betamethason as the equivalent of natural cortisol that forces the organism to actuate enzymatic defense-repair mechanisms. Enzymes are of key importance for the homeostasis of the organism and are the most sensitive clinical markers. On the basis of the rate of change in the levels of CAP_1_ and CAP_2_ in the mother's blood, one can determine when the death of the fetus has occurred or—much more importantly—could occur, or if it is in danger of miscarriage or premature birth [11–13].

 Lastly, an important role is played by the endocrine glands themselves, in which, apart from biochemical interdependencies, a significant role is played by the biophysical effect of energy flow, which one can picture as the speed of blood flow through the organs, its viscosity, the quantitative composition of individual blood cells, and so forth. In this conceptualization, the use of adrenocorticotropin hormone (ACTH) leads to the desired production of corticosteroids and their secretion by the adrenal glands but controlled in their function by the rest of the organism [14–17].

 Endogenic steroid hormones do not possess the unnatural side effects of their synthetic analogs (e.g., dexamethason or betamethason) which must, due to their prolonged effect, cause unwanted side effects through blocking endogenic production of natural corticoids. Even the administration of natural corticoids, in pharmacological doses, not only limits their endogenic production but also reciprocally slows the secretion of hormones stimulating the adrenal gland, where they originate. Similarly, apart from acting upon the maturation of fetal lungs, these medications periodically suppress the axis of hypothalamic-pituitaryglands [18–29]. Despite repeated and well-documented studies, no better dosage was found than a one-time therapy between 28 and 34 weeks and twenty four hours before birth, as proposed by Liggins and Howie [30]. It is not recommended also in cases of multiple gestations, premature rupture of membranes or fetal and maternal complications (i.e., resp, diabetes hypertension, or infection) [31–33]. The mentioned factors do not eliminate the ACTH-depot therapy, which is safe and can be used multiply during all trimesters of pregnancy. What is more, a correlation between serial administration of corticotropin and the mass, maturity, and the fetal age of the newborn has been repeatedly shown to exist. In effect, this proves the superiority of endogenic corticoids over exogenic ones in the prevention of illness and death of the newborn for high-risk pregnancies, while the usage of ACTH-depot, which additionally has no time limitations as opposed to dexamethason or betamethason. Thousands of prospectively observed pregnancies and births showed a lowered mortality count due to breathing disorders of the newborn, which has been further proven through prospective studies showing first a lessening then a complete elimination of mortality in cases of neuroendocrinologically induced hypertension as a result of the pregnancy [11, 14, 34–36].

## 2. Indication for ACTH-Depot Therapy

Indications for the treatment of pregnant women with adrenocorticotropin with a lengthened effect (ACTH-depot) are the following clinical diagnoses: neurohormonal hypothalamic postpregnancy syndrome [25], habitual miscarriages, a premature childbirth, shortened or nonexistent lactation after previous childbirths, and long-term usage of anticonception pills (especially during maturation years), as well as cytologically or colposcopically determined precancerous cervical states. Special group for the treatment are pregnant women who underwent infertility treatment, of which 67% show clinical and laboratory indication for its implementation [37–54].

 A low concentration of ACTH in the mother's blood decides on the necessity of a substitution ACTH-depot treatment. As in all hormonal examinations, the tendencies, of a rise or fall of the level of ACTH in the blood are essential. The fall of ACTH concentration is a natural occurrence only before birth in pregnancies brought to term physiologically, while at an earlier time it signals an endangerment of the pregnancy due to a miscarriage or premature birth. An administration of exogenic ACTH-depot supplements deficiencies and counters an excessive secretion of adrenocorticotropin-realising hormone (CRH), and thus extends the pregnancy. Apart from determining the level of ACTH, it is important to measure the concentration of oxytocinases (CAP_1_ and CAP_2_), the syntheses of which increases under the influence of a heightened secretion of hypothalamic hormones during pregnancy.

 ACTH dosage applies to intramuscular injection of 0.5 mg ACTH-depot, causing a 32-hour rise in the concentration of adrenal gland hormones in the mother's blood. In the case of an absence of normalization of the ACTH level in the blood and/or the persistence of clinical symptoms, the next dose can be administered already after 48 hours. Usually, a single dose in weekly intervals during the first trimester is enough, with simultaneous substitution with progesterone in physiological doses.

 There are two types of dosage of 0.5 mg ACTH-depot per dose: (1) once a week in weekly intervals or (2) a series of three doses every second day as long as the early pregnancy is endangered, either by persisting nausea, morning sickness, and pain in the abdomen or, in its later stages, by uterine cramps and bleeding, yet always by the insufficient rise of the level of oxytocinases, or, even more so, by its decline.

 The effectiveness of treatment depends primarily upon beginning the therapy as early as possible in the first stages of pregnancy, while the choice of dosage is decided by the clinical development, since the 48-hour effect of a single dose determines the need for sustaining it with a maximum of two following doses in one series. As the pregnancy develops, the almost immediate effect (disappearance of ailment) is sustained for an ever-increasing period of time. In the absence of enzymatic monitoring of the pregnancy, one may decide on the frequency of injections via the principle of ex juvantibus. Often when beginning adrenocortical therapy, one may refer to the case of hypothalamic insufficiency and not only in women with habitual miscarriages or premature deliveries. 

 Aside from enzymatic monitoring of adrenocortical therapy as the only method used in the last 40 years, currently one may determine the ACTH level in the mother's blood via laboratory procedure, optimally in the morning, or at any other daily fixed time, for best comparison of the results. Of course a level of ACTH below 5 pg/ml is an indication for a continued substitution therapy with ACTH-depot, because the hypothalamic-pituitary-adrenal axis is more significant for the viability of the fetus than the hypothalamic-pituitary-gonad axis, which was the only one taken into account until now, especially in early pregnancy still without placental steroidogenesis. The role of ACTH in creating a tolerance for the embryo becomes apparent in a slight decrease in prepregnancy level of this hormone in women with 14.1 ± 7 pg/ml to 12 ± 6 pg/ml and a return to them in the second trimester (15.4 ± 5 pg/ml) to increase in the third trimester to the highest prebirth levels of 23 ± 1 pg/ml, which, in contrast to oxytocinases, sharply decrease already during delivery. Every quick increase of the ACTH level in an early pregnancy indicates danger and requires a series of ACTH doses as a complementary therapy and is usually effective. For that reason in women who habitually miscarry, an ACTH blood level of ≥20 pg/ml is an exceptional indication for this therapy, similar to women after artificial insemination with prepregnancy levels of ≤10 pg/ml.

## 3. Exemplary Results of Adrenocortical Therapy

Table 1 shows the results of the first and second measurement of ACTH, CAP_1_, and CAP_2_ in the sixth and ninth weeks of pregnancies after infertility treatment in 24 pregnant women who miscarried, as well as in 136 pregnant women who delivered without complications, hospitalized in the first trimester in the period of ±3 weeks from the date of miscarriage of each woman from the first group. In contrast to the statistically characteristic rise of concentration levels of hormones and enzymes in the normal delivery group one cannot observe this in the group of women who miscarried [28]. The ACTH plasma concentration was assessed in whole blood samples, collected approximately at 9 o'clock in the morning in silicon-coated glass tubes containing EDTA as an anticoagulant, and centrifuged immediately in a refrigerated centrifuge. All samples were frozen at −20°C until the ACTH analysis was performed. Immunoassay was used to measure ACTH (Immulite 2000 ACTH, DPC Ltd-US). The CAP serum activity was measured by the method of Tuppy and Nesvadba, modified by Klimek in pH 7.6 (CAP_1_) and 6.9 (CAP_2_) buffer using L-cystine-di-*β*-naphthylamide as a substrate [3–5]. Statistical calculations were performed using Statistica computer program (Stat Soft, Poland). The normal distribution of values was checked by means of the Shapiro-Wilk test. Student's *t* test was applied to compare the differences between parametric data. A value of *P* < 0.05 was considered as significant.

 In the course of enzymatic monitoring of hormonal treatment of subsequent pregnancies, the levels of both CAP_1_ and CAP_2_ are lower than the initial values from previous pregnancies. Levels of CAP_1_ below 0.8 *μ*mol/L/min and CAP_2_ below 1.4 *μ*mol/L/min in early pregnancy under 10 weeks are an indication for beginning the therapy with single 0.5 mg doses of ACTH-depot, while levels of both these enzymes ≤4 *μ*mol/L/min in the third trimester require their continued use. This especially applies to laboratory-monitored pregnancies after fertilization in vitro already from the first weeks of the pregnancy and not only in those with a significantly low level of ACTH, in which the treatment with this hormone is the method of choice, but also in the case of an absence of an insignificant physiological drop in the level of this hormone and compulsory with its rising levels in the first trimester instead of only in the third [27].

 Fetal maturation is a natural time-spatial process and full-mature newborn may be small, average, or large as well as its growth may be slow, regular, or fast. Fetal maturation can be determined by the number of technical quanta of maturity, just as the body weight is measured in grams, height in centimeters and gestational age in days or weeks. Technical quantization is based on six features of the child: the position of the limbs, elbow angle, its mobility, breast nipple, plantar creases, and lanugo. Each of these features is given 2 points when fully developed or 0 points when not developed at all. It means that immature newborns have less than 6 points of thus-selected technical quanta. Maturity assessed in this way immediately after the birth found a clinical application [20–23, 53, 54]. The results of causal ACTH-depot therapy can be demonstrated with the results of two groups of pregnant women with a single fetus in a 10-year interval after a successful treatment of infertility, in other words patients characterized by a shortening of their average pregnancy duration and unfortunately doubling the numbers of births via cesarean section. In both time intervals (I:1991-1992 and II:2001–2004) 441 respectively 324 pregnant women were hospitalized, to be observed and give birth under the same clinical conditions. The examination was extended to all women consecutively hospitalized after the treatment of infertility, due to which two control groups of 161 and 182 were created which did not receive adrenocortical therapy [11, 13, 24, 54]. 

 In the assessment of clinical material, it is essential to compare both control groups, the characteristics of which coincide as shown in Table 2 separately for both examination periods. This data validates the results of previous studies concerning pregnancies after a successful treatment of infertility, for example, in the relation to the mean duration of the pregnancy (272.5 and 272.6 days), average mass (around g), the length of the newborn (54.3 cm), their advanced fetal maturity on the Klimek scale (index of 8.8 and 10.5), and the postnatal adaptation on the Apgar scale (9.7 and 9.5 points). Also to be noted are the very similar levels of CAP_1_ and CAP_2_, a good sign for both the state of the enzymatic marking methods and its diagnostic usefulness due to an exceptional (in enzymatic examinations) repeatability of results. The higher the concentration of CAP_1_ and CAP_2_ at the end of the pregnancy, the higher the neurosecretive and immunological capacity of the mother. This is confirmed by the juxtaposition of prenatal levels of these enzymes together with the corresponding increase in fetal age, mass, length, maturity, and the postnatal adaptation of the infant to the values characterizing the infants of the control groups which did not need a substitutive adrenocortical therapy.

 Compared to patients of the control groups not in need of this therapy, the patients who were treated hormonally (with the same number of births) differed only in having twice the number of miscarriages (0.87 ± 0.56 : 0.4 ± 0.8, *P* < 0.001). The application of single doses once a week extends the length of a pregnancy by an average of 8 days and increases the mass by 450 g and the length of the infant by 4 cm. The increase of the total number of doses from 4 (range 3–6) to 6 (range 1–19) causes an increase in the levels of oxytocinases in the last month of the pregnancy. 

 After ten years, the percentage of spontaneously initiated births in the control group has doubled from 39% to 78% with a simultaneous fall of the percentage of cesarean section from 39% to 21%, with an almost unchanged necessity of adding a cervical cerclage, namely 13% to 11% (Table 3).

 A slightly increased number of doses of ACTH-depot, respectively, from roughly 3.4 to 5.2 and 6.0 to 9.1 led to a halved number of cervical cerclages (from 31% to 14% and from 12% to 5%), while the number of spontaneously initiated births and cesarean section resembled control group.

 The application of ACTH-depot results in the disappearance of symptoms of a premature birth without the need for tocolysis and leads to the decrease of breathing disorders in infants. Klimek's maturity index shows a high positive correlation with fetus age, mass, and the length of the infant, while its limit value of six points statistically differentiates the maturity of the fetus [50].  Table 4 shows a comparison between the state of the newborn of same fetal maturity when the endangered pregnancy was treated with either ACTH-depot or tocolysis. The use of ACTH-depot statistically lengthens the pregnancy time by 14 days and increases the body mass by 400 g in 264 fully mature newborn infants (10–12 K points) while in 69 less mature children (6–12 K points) the pregnancy time extension is 35 days and the mass increases by 1300 g. It also eliminates premature delivery in 209 pregnant women with tocolysis where in 46 (23%) of them premature birth has been identified. A low prenatal concentration of oxytocinases on the order of 3 ± 1 *μ*mol/L/min unambiguously points to an insufficiency in the production of neurohormones. A substitutive therapy with ACTH-depot causes a normalization of the prenatal concentration of oxytocinases (7.8 and 8.1 *μ*mol/L/min). 

## 4. Conclusion

The pregnant patients in need of substitution therapy with a clinical endangered pregnancy miscarried in a whole 80% of cases before the introduction of ACTH-depot therapy in obstetrics 40 years ago [1, 6, 25]. The best results are obtained in habitual miscarriages due to the hypothalamic insufficiency of the mothers because the damage to the neurosecretory brain cells is permanent, and only the substitutive administration of ACTH is an effective procedure. Every patient should be informed about this, whether their pregnancy ended with a successful birth or a miscarriage. After giving birth in a pregnancy treated with the help of ACTH-depot, many patients mistakenly consider themselves as permanently cured of infertility.

ACTH-therapy is decisive in high-risk pregnancies, especially so in multifetal ones. The effectiveness of an enzymatically monitored therapy from the 25th week of pregnancy on is dependant in large measure on monitoring the maturity level of each fetus separately. In the case of an uneven rate of growth even when the levels of both CAP_1_ and CAP_2_ are correct, it is indicated to increase the frequency of ACTH-depot dosage.

 Pregnant women who received only single doses of the ACTH-therapy for the entire duration of pregnancy had statistically significant longer gestation and higher newborn mass and length than patients who received a series of three hormonal injections.

## Figures and Tables

**Table 1:** The results of the first laboratory examinations of women with miscarriages and births.

Pregnancy measurement	Miscarriage		Birth	
	First	Second	First	Second
Week of pregnancy	5,7 ± 1,4	9,06 ± 1,9*	6,0 ± 1,8*	9,1 ± 1,9
ACTH pg/ml	11,4 ± 3,02	14,1 ± 5,2	12,6 ± 6,2*	13,0 ± 5,2
CAP_1_ *μ*mol/L/min	0,61 ± 0,21	0,62 ± 0,13	0,64 ± 0,17*	0,92 ± 0,28
CAP_2_ *μ*mol/L/min	1,24 ± 0,44	1,39 ± 0,18	1,40 ± 0,29*	1,63 ± 0,28

*Statistically significant differences.

**Table 2:** Results of treatment of infertile women in periods I (1991–1992) and II (2001–2004) with single and serial doses from the beginning of the pregnancy.

Group	Period		Serial doses		Single doses		Control	
		*N*		*N*		*N*		*N*
Age (years)	I	441	29,9 ± 5,2	140	30,2 ± 4,9	140	29,8 ± 5,1	161
	II	324	29,6 ± 4,3	142	31,0 ± 4,6	100	30,0 ± 5,1	182
Number of pregnancies	I		3,2 ± 1,3		2,7 ± 1,5		2,0 ± 1,3	
	II		1,9 ± 1,3		2,0 ± 1,2		1,8 ± 1,3	
Number of births	I		2,3 ± 1,1		1,4 ± 0,7		1,6 ± 0,8	
	II		1,2 ± 0,6		1,2 ± 0,6		1,4 ± 0,8	
Number of miscarriages	I		0,9 ± 1,1		1,3 ± 1,4		0,4 ± 0,8	
	II		0,7 ± 1,0		0,8 ± 0,9		0,4 ± 0,8	
Number of children	I		0,7 ± 0,8		1,2 ± 0,5		1,3 ± 0,6	
	II		1,2 ± 0,5		1,2 ± 0,6		1,4 ± 0,7	
Number of doses	I		3,4 ± 0,6		6,0 ± 1,5		0	
	II		5,2 ± 2,8		9,1 ± 3,5		0	
CAP_1_ *μ*mol/L/min	I		6,6 ± 2,1		8,1 ± 2,3		8,8 ± 2,4	
	II		7,7 ± 2,5		7,8 ± 2,5		8,8 ± 2,4	
CAP_2_ *μ*mol/L/min	I		5,1 ± 1,5		6,6 ± 1,7		7,1 ± 1,7	
	II		6,4 ± 1,8		6,5 ± 1,8		6,8 ± 1,9	
Length of pregnancy (days)	I		262,5 ± 32		267,6 ± 11		272,5 ± 11	
	II		270,7 ± 22		275,0 ± 11		272,6 ± 12	
Mass of newborn (g)	I		2895 ± 900		3165 ± 440		3299 ± 439
	II		3230 ± 504		3477 ± 420		3341 ± 480
Length of newborn (cm)	I		50,5 ± 7,2		53,4 ± 2,7		54,3 ± 2,7	
	II		53,6 ± 3,2		55,0 ± 2,5		54,4 ± 2,6	
Apgar scale points	I		9,7 ± 0,7		9,7 ± 1,6		9,5 ± 0,8	
	II		9,5 ± 0,8		9,7 ± 0,9		9,7 ± 1,7	
Klimek scale points	I		9,8 ± 1,6		9,9 ± 1,6		10,5 ± 1,4	
	II		8,8 ± 1,6		8,9 ± 1,7		8,8 ± 1,7	

**Table 3:** Clinical characteristics of examined groups in the first (I) and second (II) periods.

Group	Period	Serial doses		Single doses		Control	
		*N*	(%)	*N*	(%)	*N*	(%)
Cervical	I	43	(31%)	17	(12%)	21	(13%)
cerclage	II	20	(14%)	5	(5%)	20	(11%)
Spontaneous	I	116	(83%)	86	(61%)	54	(39%)
birth	II	101	(71%)	82	(82%)	142	(78%)
Cesarean	I	21	(13%)	63	(39%)	63	(39%)
section	II	32	(23%)	25	(25%)	38	(21%)

**Table 4:** Comparison of the state of the newborn according to the reached level of maturity depending on the use of ACTH-depot or tocolysis in the examined pregnancy.

Klimek's maturity index	12–10		9–6		Below 6	
ACTH-therapy	Yes	No	Yes	No	Yes	No
New-born (*n* (%))	264 (80%)	89 (45%)	69 (20%)	63 (32%)	0	46 (23%)
Fetus age (days)	273 ± 10	259 ± 14	266 ± 12	231 ± 10		175 ± 17
Mass (kg)	3,4 ± 0,4	3,0 ± 0,6	3,3 ± 0,5	2,0 ± 0,5		1,2 ± 0,3
Maturity (points)	11,4 ± 0,7	11,0 ± 0,5	8,2 ± 0,9	8,0 ± 1,0		4,0 ± 1,0
Apgar adaptation	9,6 ± 0,8	10,0 ± 1,0	9,4 ± 0,8	8,0 ± 1,0		5,0 ± 2,0
Oxytocinase (*μ*mol/L/min)	7,8 ± 2,8	3,0 ± 0,7	8,1 ± 3,4	3,0 ± 1,0		3,0 ± 1,0
